# Anti-*Kp^a^* antibody: Getting to know a strange and dangerous specificity

**DOI:** 10.1016/j.htct.2026.106269

**Published:** 2026-03-12

**Authors:** Marcela Quintero Santacruz

**Affiliations:** Universidad del Valle, Cali, Colombia

**Keywords:** Alloantibodies, Red blood cells, Antigens, Hemolytic transfusion, Hemolytic disease

## Abstract

Anti-*Kp^a^* is an irregular antibody of clinical significance directed against the red blood cell antigen *Kp^a^* of the Kell system. It is rare in the general population and, therefore, uncommon as a cause of transfusion or hemolytic complications. It has been documented in isolated cases, mainly in the context of alloimmunization after transfusion exposure or during pregnancy; its incidence in clinical practice is limited and can be difficult to detect in routine pre-transfusion testing, which could lead to failure to identify the antibody prior to transfusion.

The production of Anti-*Kp^a^* antibodies is less common than antibodies against other Kell system antigens, such as anti-K. However, when generated, these antibodies can mediate hemolytic reactions in patients receiving incompatible blood and can cause hemolytic disease in the fetus since in addition to immune destruction of red blood cells, Kell system antibodies such as Anti-*Kp^a^* can cause suppression of fetal erythropoiesis, resulting in severe anemia.

## Introduction

Erythrocyte alloimmunization is a complex immunological process that occurs in patients who have been exposed to foreign erythrocyte antigens, either through blood transfusions or gestation, and who develop irregular antibodies against these antigens [1]. This phenomenon is particularly relevant in patients who require repeated transfusions, such as those with chronic anemia, as the presence of clinically significant antibodies can complicate transfusion management and increase the risk of hemolytic reactions [1].

The Anti-*Kp^a^* antibody is a rare specificity within the Kell blood group system. Due to its low prevalence in the general population, it is an infrequent cause of hemolytic transfusion reactions or hemolytic complications [2]. This antibody, directed against the *Kp^a^* antigen, has been documented in isolated cases, mainly in the context of alloimmunization following transfusion exposure or during pregnancy, but its incidence in clinical practice is limited [2].

The aim of this review is to provide comprehensive and up-to-date information on the Anti-*Kp^a^* antibody and its relevance in transfusion medicine.

### Relevant characteristics of the *Kp^a^* antigen

The *Kp^a^* antigen is part of the Kell blood group system (ISBT 006). This system comprises 38 antigens carried by a 732-amino acid transmembrane glycoprotein known as CD238. The nomenclature derives from ‘Kelleher’, the first producer of an anti-K antibody, and ‘p’ for ‘Penney,’ the name of the first patient identified as an Anti-*Kp^a^* producer [3]. This antigen, first discovered in 1957, has gained notoriety due to its rarity and its involvement in transfusion reactions and hemolytic disease of the fetus and newborn (HDFN) [3].

The *Kp^a^* antigen, referred to by the International Society of Blood Transfusion (ISBT) as KEL3, is an allelic variant of the protein encoded by the KEL gene, located on chromosome 7q32-q36 [3]. This gene gives rise to the Kell glycoprotein, a metalloenzyme expressed in the erythrocyte membrane that performs several functions such as regulation of cell growth and interaction with other membrane proteins [3].

The variation in *Kp^a^* is due to a polymorphism in the KEL gene, which generates an amino acid difference in the resulting protein [3]. This makes *Kp^a^* a low frequency antigen in most populations, with an estimated prevalence of <2 % in Caucasians and virtually absent in other ethnic groups. Conversely, its antithetical partner, the *Kp^b^* antigen (KEL4), is a high-frequency antigen, appearing in >99 % of individuals [3].

Table 1 illustrates the phenotypes of the Kell system, frequency in European and African populations and the reported clinical significance:

**Table 1:** Kell system phenotypes, reported frequencies in European and African populations and their clinical significance.

Phenotype (ISBT Nomenclature)	Antigen	Reported Frequency	Clinical Significance
K:−1,2	k (006,002)	91 % European and African Populations	The most common phenotype; anti-K antibodies (006,001) can cause severe reactions.
K:1,2	K (006,001) k (006,002)	9 % European and African Populations	Compatible with most transfusions; risk with anti-K antibodies.
K:1,−2	K (006,001)	0.2 % European and African Populations	Rare phenotype; can cause significant incompatibility due to the absence of k
Kp(*a* + *b*-) (K:−3,4)	Kpᵃ (006,003)	0.1 % European and African Populations	Very rare phenotype; anti-Kpᵇ antibodies (006,004) may be clinically significant.
Kp(a-*b*+) (K:3,−4)	Kpᵇ (006,004)	97.9 % European and African Populations	Common phenotype; anti-Kpᵃ antibodies are important in transfusion medicine.
Kp(*a* + *b*+) (K:3,4)	Kpᵃ (006,003) Kpᵇ (006,004)	2 % European and African Populations	Compatible with most transfusions.
Js(*a* + *b*-) (K:−5,6)	Jsᵃ (006,005)	0.01 % European; 20 % African	Very rare in Europe; common in populations of African descent. Anti-Jsᵇ (006,006) antibodies are relevant.
Js(a-*b*+) (K:5,−6)	Jsᵇ (006,006	81 % African	Anti-Jsᵃ antibodies may cause hemolytic reactions, especially in Africans.
Js(*a* + *b*+) (K:5,6)	Jsᵃ (006,005) Jsᵇ (006,006)	19 % African	Compatible with some donors, but there may be incompatibility in specific cases.

Information on the frequencies of KEL:3,4 phenotypes in patients with hemoglobinopathies and blood donors in South America is scarce due to the rarity of these specific variants in the general population and the limited specific genetic research in this region [3,4]. Thus, genetic studies focused on local populations, especially in polytransfused patients and blood donors, is necessary [3,4].

### Relevant characteristics of anti-*Kp^a^*

The low frequency of the *Kp^a^* antigen reduces the likelihood of sensitization in the general population [4]. However, when it does occur, the formation of anti-*Kp^a^* antibodies can have serious clinical implications [4]. Sensitization to *Kp^a^* occurs primarily through blood transfusions or, less frequently, during pregnancy when a *Kp^a^*-negative mother is exposed to *Kp^a^*-positive fetal red blood cells [4].

The production of anti-*Kp^a^* is less common than that of other Kell system antibodies, such as anti-K. The latter is known to suppress erythropoiesis by an immunologic mechanism that affects both the production of erythroid progenitors in the bone marrow and the survival of mature erythrocytes in the circulation [4]. Anti-K can bind to erythroid progenitor cells expressing the Kell antigen in the bone marrow and trigger mechanisms such as macrophage-mediated phagocytosis or functional interference where antibodies block signals essential for the proliferation and maturation of these cells, directly affecting erythropoiesis [4].

Alloimmunization against the *Kp^a^* antigen is extremely rare, even in polytransfused patients, such as those with hemoglobinopathies [5]. Anti-*Kp^a^* can mediate hemolytic reactions in patients receiving incompatible blood by causing extravascular hemolysis through opsonization of erythrocytes leading to destruction in the reticuloendothelial system primarily in the spleen and liver [5]. Fortunately, due to the low prevalence of *Kp^a^*, the risk of developing such antibodies and having a transfusion reaction is relatively low compared to other blood systems [5].

Anti-*Kp^a^* can be difficult to detect in routine pre-transfusion testing, which could lead to failure to identify the antibody prior to transfusion [5]. In addition to immune destruction of red blood cells, anti-*Kp^a^* can possibly cause suppression of fetal erythropoiesis with consequent severe anemia as reported by Tuson et al. where the first case of probable erythropoiesis suppression attributable to anti-*Kp^a^* is described in a twin pregnancy where only one of the twins was affected [6].

Anti-*Kp^a^* is an immunoglobulin G (IgG), predominantly of the IgG1 subtype. While naturally occurring antibodies are extremely rare, they have been reported in a 12-month-old infant with recurrent infections as a cross-reaction to bacterial capsule antigens [7].

### Case reports related to hemolytic disease of the fetus due to anti-*Kp^a^*

Alloimmunization against the *Kp^a^* antigen is extremely rare; the medical literature documents very few cases of perinatal anemia or miscarriages associated with anti-*Kp^a^* antibodies [3, 4, 5]. Most studies and reports focus on antibodies against other Kell system antigens, such as K1 (KEL1), due to their higher prevalence [3, 4, 5].

The 2013 report by Rossi et al. documents a case of HDFN caused by the anti-*Kp^a^* antibody [8]. In this case, a pregnant woman developed anti-*Kp^a^* antibodies resulting in severe fetal anemia. The diagnosis was confirmed by serologic testing and prenatal imaging studies that revealed signs of hydrops fetalis, a severe complication in HDFN. This antibody was initially identified as part of an investigation for parvovirus B19-induced fetal anemia. Clinical management included multiple intrauterine transfusions to correct the fetal anemia, which allowed the pregnancy to be prolonged to a point where delivery was viable. The newborn, although affected by anemia, was stabilized after birth, but required additional monitoring and treatment. This case underscores the importance of early detection and proper management of alloimmunization by antibodies such as anti-*Kp^a^*, because of the possibility of serious consequences if not treated early [9]. However, the paucity of specific reports makes it difficult to accurately assess the frequency and severity [9].

### Erythrocyte phenotyping and transfusion impact

Extended erythrocyte phenotyping facilitates the identification of anti-*Kp^a^* in patients requiring transfusion. This is particularly critical for polytransfused patients and women of childbearing age, as alloimmunization within the Kell system can significantly impact future pregnancies [10]. In the laboratory, identification of an anti-*Kp^a^* antibody is carried out through serological testing, using techniques such as the indirect antiglobulin test (IAT) [10]. In cases where serological analysis is inconclusive, genetic testing may be useful to detect polymorphisms in the KEL gene [10].

In transfusion situations, it is suggested to administer *Kp^a^*-negative red blood cell units to alloimmunized patients, although some studies suggest that serologically compatible units may also be adequate, given the low relative risk of severe hemolysis [11]. However, in scenarios such as hemolytic disease of the newborn, anti-*Kp^a^* may generate fetal anemia, although to a lesser degree than other Kell system incompatibilities [11].

For patients requiring transfusions, the general recommendation is to use blood units that are compatible by crossmatching at 37 °C in the antiglobulin phase without the need to routinely select *Kp^a^*-negative units due to the low prevalence of the antigen in the population [12]. However, in situations where anti-*Kp^a^* has been identified, for example in HDFN and sickle cell disease, it is essential to ensure strict compatibility to prevent severe complications [12].

## Conclusions

Appropriate management of patients with anti-*Kp^a^* antibodies requires an individualized approach, with special attention to the compatibility of transfused units and erythrocyte phenotyping. Alloimmunization, although rare, underlines the need to implement preventive strategies and accurate diagnosis to avoid hemolytic complications both in transfusions and in the perinatal context. This type of finding, although infrequent, highlights the relevance of detecting irregular antibodies in pregnant women, as well as the need to perform specific compatibility tests prior to transfusions to ensure the safety of both the mother and the fetus.

It is also important to perform genetic studies focused on local populations, especially in polytransfused patients and blood donors, since information on the specific variants and the frequency of alloimmunization and transfusion and gestational hemolysis due to these antibodies is scarce in the Americas.

## Data availability

The data used to support this study's findings are included within the article.

## Conflicts of interest

The author declares no conflicts of interest.
